# Protective Role of trans-Chalcone against the Progression from Simple Steatosis to Non-alcoholic Steatohepatitis: Regulation of miR-122, 21, 34a, and 451

**DOI:** 10.34172/apb.2022.022

**Published:** 2021-01-31

**Authors:** Elham Karimi-Sales, Sajad Jeddi, Abbas Ebrahimi-Kalan, Mohammad Reza Alipour

**Affiliations:** ^1^Drug Applied Research Center, Tabriz University of Medical Sciences, Tabriz, Iran.; ^2^Endocrine Physiology Research Center, Research Institute for Endocrine Sciences, Shahid Beheshti University of Medical Sciences, Tehran, Iran.; ^3^Neurosciences Research Center, Tabriz University of Medical Sciences, Tabriz, Iran.; ^4^Department of Physiology, Faculty of Medicine, Tabriz University of Medical Sciences, Tabriz, Iran.

**Keywords:** Non-alcoholic steatohepatitis, trans-Chalcone, miRNAs, Liver, Rat

## Abstract

***Purpose:*** Non-alcoholic steatohepatitis (NASH) is an inflammatory disorder and an aggressive form of fatty liver disease. Certain microRNAs, including miR-122, 21, 34a, and 451, are involved in the transition from steatosis to NASH. This study examined how trans-chalcone (the core of chalcone derivatives) affects NAFLD progression by regulating miRNAs.

***Methods:*** Male rats were divided into three groups (n = 7/group) as follows: control, rats were gavaged with 10% tween 80 (for two weeks); NASH, rats were gavaged with a high-fat liquid diet (HFD; for six weeks) and 10% tween 80 (for two weeks); NASH + Chal, rats were gavaged with the HFD (for six weeks) and trans-chalcone (for two weeks). Hepatic expression levels of miR-122, 21, 34a, and 451 were determined.

***Results:*** trans-Chalcone reversed histological abnormalities, reduced liver injury markers, and attenuated insulin resistance in HFD-fed rats. In the liver, HFD-induced NASH increased the expression level of miR-34a and decreased expression levels of miR-122, 21, and 451. However, trans-chalcone inhibited HFD-induced changes in expression levels of these miRNAs.

***Conclusion:*** trans-Chalcone could inhibit the transition from steatosis to NASH through the modulation of miR-122, 21, 34a, and 451 expression levels in the liver.

## Introduction

Nowadays, non-alcoholic fatty liver disease (NAFLD) is a well-known cause of chronic liver disease.^1-3^ The increasing prevalence of NAFLD has been linked to a combination of a sedentary lifestyle and excess calorie intake.^4-6^ This common liver disease ranges from simple steatosis (lipid accumulation in more than 5-10 percent of hepatocytes) to non-alcoholic steatohepatitis (NASH; the inflammatory and more aggressive stage), leading to liver cirrhosis and -only in a small number of patients- liver cancer.^7-10^ It has been known that inflammation, fat accumulation in the liver tissue, hepatocellular damage, and fibrosis are characteristics of NASH.^8^

MicroRNAs (miRNAs) are extremely conserved, endogenous, small, and non-coding RNAs involved in regulating gene expression using the post-transcriptional mechanisms.^11-14^ It has been known that dysregulation of specific microRNAs contributes to NAFLD progression.^13,15^ In particular, dysregulation of miR-122, 21, 34a, and 451 is responsible for transitioning from simple steatosis to NASH.^16^ Therefore, these non-coding RNAs are possible therapeutic targets for NAFLD/ NASH treatment.^15^

Lack of FDA approved anti-NASH drugs creates an urgent need to develop effective and safe medicines to treat this liver disease.^17^ Chalcone derivatives have protective effects on NAFLD and liver cancer through several molecular mechanisms.^18^ In this regard, it has been shown that *trans*-chalcone, the core structure of chalcones, protects high-fat diet (HFD)-fed rats against liver inflammation and insulin resistance by miR-34a, 451, and 33a-dependent mechanisms.^19^ Furthermore, co-administration of this chalcone with HFD improved hepatic lipid metabolism and protected rats against NASH induction.^20^ The possible effect of *trans*-chalcone on the transition from simple steatosis to NASH and its impact on expression levels of miR-122 and 21, critically involved in the NAFLD progression, is still unknown. The present study investigated how orally administered *trans*-chalcone affects NAFLD progression by regulating hepatic miR-122, 21, 34a, and 451 levels.

## Material and Methods

### Animal experiments

Male Wistar rats (210–250 grams) were obtained and kept under standard conditions (22 ± 2°C and 12 hours light: 12 hours dark cycle). Animals had free access to food (standard rat diet) and water.

After acclimatization period (one week), twenty-one rats were divided randomly into three experimental groups and kept for six weeks (n = 7/group) as follows: (1) control group, received 10% tween 80 (2 mL);^19,21^ (2) NASH group, received a high-fat liquid diet (HFD, 10 ml/kg)^22^ and 10% tween 80; (3) NASH + Chal group, received the HFD and *trans*-chalcone (20 mg/kg dissolved in 2 mL 10% tween 80).^19^ Rats received HFD by oral gavage at 6:00 p.m. once a day for six weeks. Moreover, *trans*-chalcone (Sigma-Aldrich, Germany, Product Number: 136123, 97%) or 10% tween 80 was administered by oral gavage at 8:00 a.m. once a day for two weeks (from fifth to sixth week). Treatment with *trans*-chalcone was performed after induction of simple steatosis. Two rats were gavaged with HFD for four weeks to confirm the induction of simple steatosis by four weeks of HFD feeding. At the end of treatments, overnight fasted (8 hours) rats were sacrificed under deep anesthesia.^19^ Then, blood samples were collected, livers photographs were taken by a digital camera (Canon EOS 6D, Japan), and liver samples were removed. Liver samples were frozen for gene expression assay or fixed at 10% neutral buffered formalin for histological studies.

### Biochemical measurements

Serum levels of liver injury markers, including alkaline phosphatase (ALP), aspartate aminotransferase (AST), and alanine aminotransferase (ALT)^23^ were measured employing commercial kits (Pars Azmoon, Tehran, Iran). Fasting serum insulin concentration was determined through a rat specific insulin ELISA kit (Mercodia, Uppsala, Sweden). Moreover, fasting plasma glucose level was measured by a digital glucometer (Gluco Sure, Star, Taiwan). Then, insulin resistance was estimated by calculation of the homeostasis model assessment parameter of insulin resistance (HOMA-IR) through the following formula^24^:

HOMA-IR = fasting serum insulin (µU/m mL) × fasting plasma glucose (mmol/L) / 22.5

### Real-time polymerase chain reaction (PCR)

Real-time PCR was done to measure the expression levels of miR-122, 21, 34a, and 451 in the liver samples. In this regard, total RNA extraction and complementary DNA synthesis steps were done as previously described.^21^ SYBR Green PCR Master Mix (Fermentas, Germany) was used in real-time PCR reactions.^21^ Amplifications were performed in a rotor gene 6000 real-time PCR machine (Corbett, Life science, Sydney, Australia). Target genes were normalized with miR-191 (housekeeping gene). Finally, the 2 ^−(ΔΔCt)^ method was used to calculate the relative quantitative expression level of each target miRNA.^21^ Sequences of primers are shown in Table 1.

**Table 1:** Sequences of primers

**Gene name**	**Accession number**	**Target sequence** ^a^
rno-miR-122-5p	MIMAT0000827	UGGAGUGUGACAAUGGUGUUUG
rno-miR-21-5p	MIMAT0000790	UAGCUUAUCAGACUGAUGUUGA
rno-miR-34a-5p	MIMAT0000815	UGGCAGUGUCUUAGCUGGUUGU
rno-miR-451-5p	MIMAT0001633	AAACCGUUACCAUUACUGAGUU
rno-miR-191-5p	MIMAT0000866	CAACGGAAUCCCAAAAGCAGCUG

^a^Sequence was derived from miRBase (www.mirbase.org)

### Histological assessment

Formalin-fixed liver samples were embedded in paraffin, stained with hematoxylin and eosin, and analyzed blindly by an experienced pathologist under a light microscope (Axioskop2; Carl Zeiss MicroImaging Inc., Germany). The degree of steatosis was scored as follows: (0) no steatosis, (1) microsteatosis, (2) microsteatosis plus mild macrosteatosis, (3) high macrosteatosis. The inﬂammation was also scored as follows: (0) none, (1) mild infiltration of lymphocytes into the portal triad, (2) high infiltration of lymphocytes into the portal triad, (3) high infiltration of lymphocytes all over the liver. Hepatocyte ballooning was scored as follows: (0) none, (1) low, (2) high.^20^

### Statistical analysis

Statistical analyses were done using SPSS 16 software (SPSS Inc., Chicago, IL, USA). Analyses of data obtained by real-time PCR or biochemical measurements were performed by one-way ANOVA with Tukey’s post hoc test. These data are shown as mean ± SEM. Besides, histological data were analyzed using Kruskal-Wallis and Mann Whitney test as post hoc analysis. Histological data are expressed as median (min-max). Statistical significance was defined as a *P* value < 0.05. The graphs were plotted using GraphPad Prism 7 (GraphPad Software, Inc., La Jolla, CA, USA) software.

## Results

### trans-Chalcone and biochemical parameters

As shown in Table 2, NASH induction significantly (*P* < 0.001) increased the HOMA-IR score as well as serum levels of ALP, AST, and ALT. Conversely, *trans*-chalcone significantly (*P* < 0.001) inhibited HFD-induced changes in HOMA-IR score and serum levels of liver injury markers (ALP, AST, and ALT) in rats of NASH + Chal group compared with the NASH group (Table 2).

**Table 2:** Biochemical parameters in study groups^a^

	**Control**	**NASH**	**NASH + Chal**
HOMA-IR	6.36 ± 0.183	16.77 ± 1.595^***^	5.653 ± 0.138^###^
ALP (IU/L)	459.667 ± 13.776	933.5 ± 7.756^***^	581 ± 3.183^***,###^
AST (IU/L)	102.6667 ± 4.255	161.75 ± 1.264^***^	92.8 ± 0.771^**,###^
ALT (IU/L)	41.66667 ± 1.453	80 ± 0.598^***^	45 ± 0.471^*,###^

ALP, alkaline phosphatase; ALT, alanine aminotransferase; AST, aspartate aminotransferase; NASH, non-alcoholic steatohepatitis. Data are expressed as mean ± SEM.^*^P < 0.05 compared with the control group, ^**^ P < 0.01 compared with the control group, ^***^ P < 0.001 compared with the control group, ^###^* P* < 0.001 compared with the NASH group. ^a^Control: received 10% tween 80 for two weeks, NASH: received the high-fat liquid diet for six weeks and 10% tween 80 for two weeks, NASH + Chal: received the high-fat liquid diet for six weeks and trans-chalcone for two weeks.

### trans-Chalcone and hepatic miRNAs

**Figure 1:**
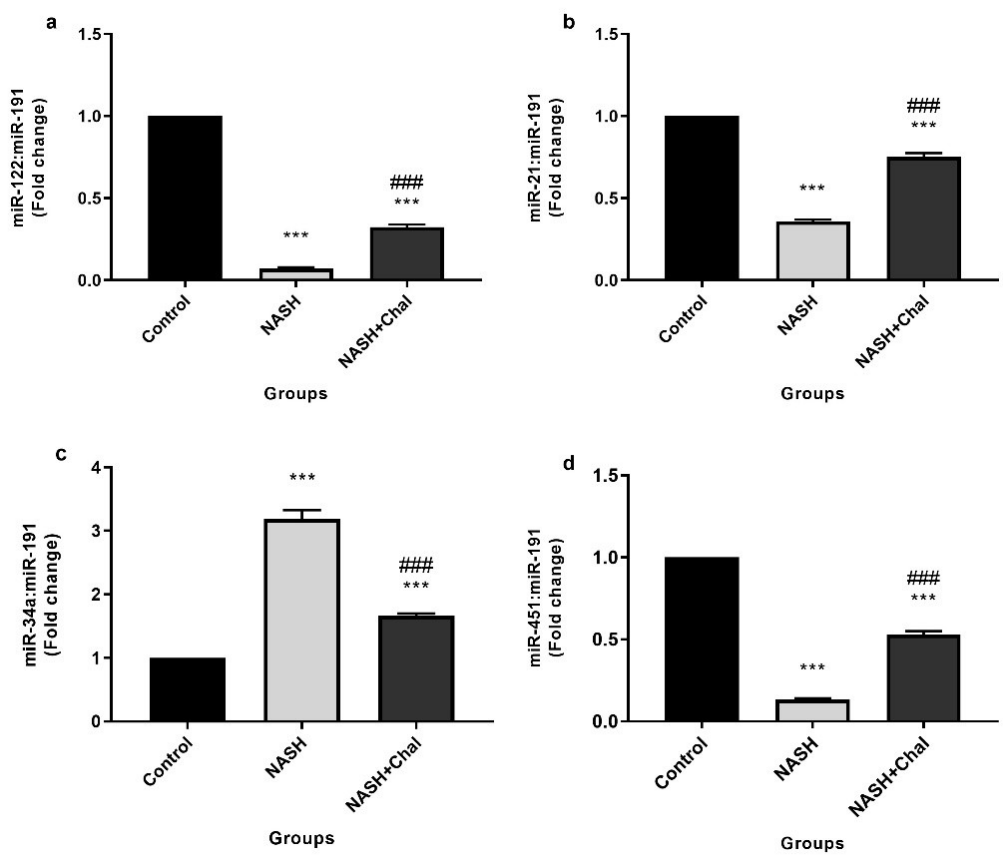
Hepatic levels of miR-122 (a), 21 (b), 34a (c), and 451 (d) in the study groups(control: received 10% tween 80 for two weeks, NASH: received the high-fat liquid diet for six weeks and 10% tween 80 for two weeks, NASH + Chal: received the high-fat liquid diet for six weeks and *trans*-chalcone for two weeks). Data are expressed as mean ± SEM. ^***^ P < 0.001 compared with the control group, ^###^*P* < 0.001 compared with the NASH group. Chal, *trans*-chalcone; NASH, non-alcoholic steatohepatitis.

Hepatic expression levels of miR-122, 21, 34a, and 451 are shown in Figure 1. Induction of NASH significantly (*P* < 0.001) decreased miR-122, 21, and 451 expression levels. Besides, expression levels of miR-34a were significantly (*P* < 0.001) higher in the NASH group than the control group. On the contrary,* trans*-chalcone significantly (*P* < 0.001) inhibited NASH-related changes in hepatic expression levels of miR-122, 21, 34a, and 451 (Figure 1 a-d).

### trans-Chalcone and NASH-related histological changes

**Figure 2:**
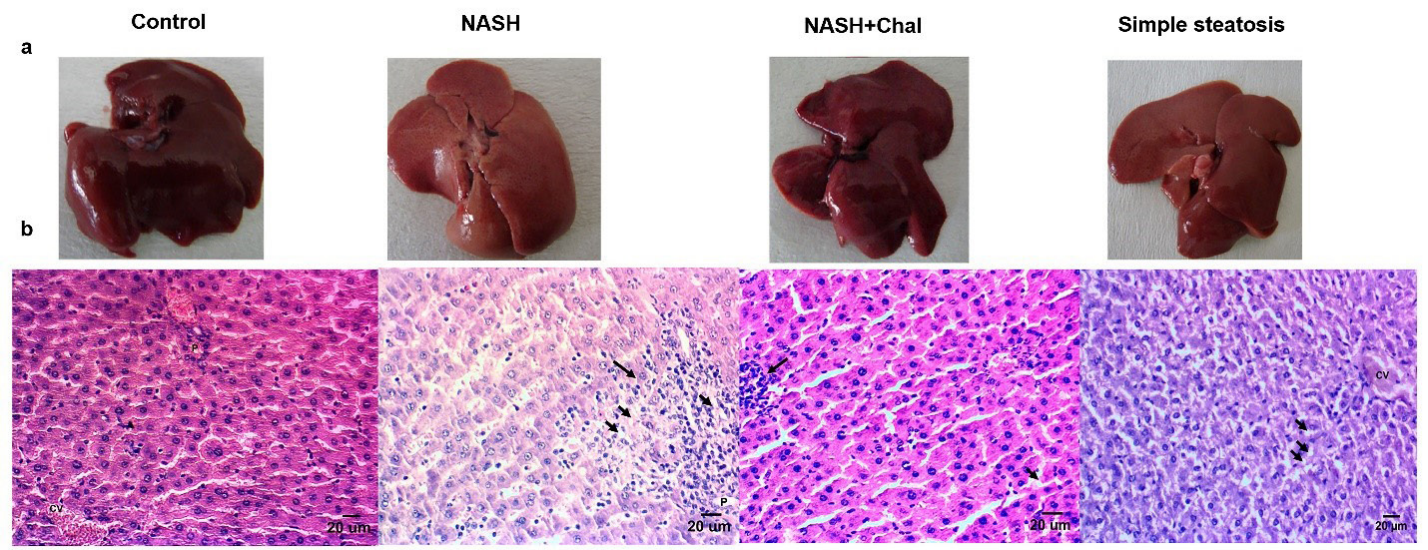
Liver morphology (a) and hematoxylin and eosin-stained liver specimens (b) in study groups (control: received 10% tween 80 for two weeks, NASH: received the high-fat liquid diet for six weeks and 10% tween 80 for two weeks, NASH + Chal: received the high-fat liquid diet for six weeks and *trans*-chalcone for two weeks). Arrows show inflammatory cells infiltrations or lipid droplets. Steatosis and severe lymphocyte infiltration were observed in the NASH group. However, these histological features of NASH were reduced in NASH + Chal group. Moreover, accumulations of lipid droplets were observed in the livers of rats with simple steatosis. Scale bar: 20 μm. Chal, *trans*-chalcone; CV, central vein; NASH, non-alcoholic steatohepatitis.

Development of simple steatosis after four weeks and NASH after six weeks of HFD feeding were confirmed by macroscopic and microscopic studies. In this regard, the livers of rats with NASH and simple steatosis became yellow. However, *trans*-chalcone protected the livers of rats in the NASH + Chal group against this color change (Figure 2a). In the histological studies, livers of rats with simple steatosis showed lipid droplets accumulations. Also, livers of rats in the NASH group showed accumulations of lipid droplets, severe infiltration of lymphocytes, and hepatocellular ballooning. On the contrary, *trans*-chalcone reversed NASH-related histological changes in the NASH + Chal group compared with the NASH group (Table 3 and Figure 2b).

**Table 3:** Histological results of study groups^a^

	**Control**	**NASH**	**NASH + Chal**
Steatosis	0 (0-1)	2 (2-3) ^*^	1 (1-2) ^#^
Inflammation	0 (0-1)	2 (1-3) ^*^	1.5 (1-2)
Ballooning	0 (0-0)	2 (0-2)^*^	1 (0-2)

Chal, *trans*-chalcone; NASH, non-alcoholic steatohepatitis. Values are expressed as median (min-max). *P < 0.05 compared with the control group, ^#^*P* < 0.05 compared with the NASH group. ^a^Control: received 10% tween 80 for two weeks, NASH: received the high-fat liquid diet for six weeks and 10% tween 80 for two weeks, NASH + Chal: received the high-fat liquid diet for six weeks and *trans*-chalcone for two weeks.

## Discussion

In this study, two weeks of treatment with *trans*-chalcone could inhibit the transition from simple steatosis to NASH in rats fed the HFD for six weeks. This chalcone reduced serum levels of liver injury markers (ALP, AST, and ALT) and also improved histological abnormalities in the livers of HFD-fed rats, including lipid accumulation, infiltration of lymphocytes, and hepatocellular ballooning. Consistent with these findings, a recent study indicated that co-administration of *trans*-chalcone with HFD for six weeks could protect HFD-fed animals against NASH induction.^20^

Insulin resistance is strongly linked to severe forms of NAFLD.^25^ In the current study, *trans*-chalcone inhibited insulin resistance in HFD-fed rats. Consistent with this finding, the protective effects of this chalcone against hyperglycemia, hyperinsulinemia, and insulin resistance have been suggested by previous studies.^19,26^ Hence, it seems that *trans*-chalcone has a protective role against NAFLD progression by its inhibitory effect on insulin resistance.

The progression of NAFLD/ NASH is linked to the altered hepatic levels of specific miRNAs. Dysregulation of these miRNAs can affect inflammation, insulin signaling, response to tissue injury, and lipid metabolism.^27^ In the current study, *trans*-chalcone had a hepatoprotective role against NAFLD progression by inhibiting changes in the hepatic expression levels of miR-122, 21, 34a, and 451.

It has been known that miR-122, a liver-specific miRNA, accounts for 70% of the total miRNA pool in the liver.^28^ Loss of this miRNA in knockout mice results in lipid accumulation, inflammation, fibrosis, and finally, the development of hepatocellular carcinoma.^29^ In the present study, the expression of hepatic miR-122 was lower in the NASH group. However, *trans*-chalcone could inhibit this HFD-induced change. Consistent with this study, Cheung et al^27^ indicated that NASH patients had decreased miR-122 levels. Moreover, in a study by Yang et al,^30^ reduction of miR-122 led to insulin resistance, and licorice flavonoid could reverse this change.

In another previous study, HFD increased the hepatic expression levels of miR-122. In contrast, lychee pulp phenolics ameliorated HFD-induced change in miR-122 expression and alleviated steatosis in the liver of HFD-fed animals.^31^ Similarly, proanthocyanidins could reverse the up-regulation of miR-122 in dyslipidemic obese rats.^32^ Therefore, it seems that miR-122 has different roles in HFD-induced dyslipidemia/ simple steatosis and NASH conditions. In the present study, HFD-induced NASH was linked to the down-regulation of hepatic miR-122 levels. However, *trans*-chalcone markedly inhibited this HFD-induced change.

In this study, NASH induction was associated with decreased expression levels of miR-21. There are controversial results about the link between HFD feeding and hepatic miR-21 levels. Blasco-Baque et al^33^ indicated that miR-21 expression was negatively correlated with the hepatic triacylglycerol content during metabolic adaptation of the liver to HFD. Furthermore, Ahn et al^34^ suggested that lycopene could inhibit liver steatosis through the up-regulation of hepatic miR-21. However, a previous study indicated that this miRNA is overexpressed in NASH condition.^35^ It seems that the hepatic expression level of miR-21 is dependent on both animal model and diet,^33^ and *trans*-chalcone could inhibit its pathologic decrease in NASH.

The next part of the present study suggested the ability of *trans*-chalcone to reverse HFD-induced up-regulation of hepatic miR-34a in rats with NASH. Similarly, the inhibitory effects of *trans*-chalcone on hepatic expression levels of this miRNA in normal and HFD-fed rats were observed in recent studies.^19,21^ On the other hand, miR-34a is overexpressed in NASH, steatosis, and insulin resistance.^5,27,36^ A previous study by Choi et al^37^ suggested that overexpression of this miRNA in the liver tissue caused obesity-mimetic consequences. Conversely, antagonism of miR-34a could inhibit liver inflammation and glucose intolerance in obese animals.^37,38^

Furthermore, dysregulation of miRNA-451 is involved in NASH development.^39^ In the current study, NASH development was linked to the down-regulation of this miRNA, and *trans*-chalcone could inhibit this alteration. In this regard, the reductions of hepatic expression levels of miR-451 were observed in NASH patients and a mouse model of HFD-induced NASH.^39^ It seems that miR-451 negatively regulates inflammation and gluconeogenesis in the liver.^39,40^ Zhuo et al suggested that overexpression of hepatic miR-451 improved glucose intolerance in HFD-fed animals.^40^ Moreover, this miRNA negatively regulated the production of the inflammatory cytokines.^39^ It seems that the anti-NASH effect of *trans*-chalcone is partly mediated by inhibition of the HFD-induced changes in hepatic levels of miR-34a and 451.

## Conclusion

Protective role of *trans*-chalcone against the progression form simple steatosis to NASH is linked to the ability of this chalcone to inhibit dysregulation of miR-122, 21, 34a, and 451 in the liver of HFD-fed animals.

## Ethical Issues

All animal experiments were approved by the Ethics Committee of Tabriz University of Medical Sciences (Approval ID: IR.TBZMED.VCR.REC.1397.278).

## Conflict of Interest

All authors stated no conflicts of interest.

## Acknowledgments

This study was funded by the Vice Chancellor for Research of Tabriz University of Medical Sciences, Tabriz, Iran [grant number: 61562].

## References

[1] Shojaei Zarghani S, Abbaszadeh S, Alizadeh M, Rameshrad M, Garjani A, Soraya H (2018). The effect of metformin combined with calcium-vitamin D3 against diet-induced nonalcoholic fatty liver disease. Adv Pharm Bull.

[2] Asghari S, Rafraf M, Farzin L, Asghari-Jafarabadi M, Ghavami SM, Somi MH (2018). Effects of pharmacologic dose of resveratrol supplementation on oxidative/antioxidative status biomarkers in nonalcoholic fatty liver disease patients: a randomized, double-blind, placebo-controlled trial. Adv Pharm Bull.

[3] Younossi ZM, Stepanova M, Afendy M, Fang Y, Younossi Y, Mir H (2011). Changes in the prevalence of the most common causes of chronic liver diseases in the United States from 1988 to 2008. Clin Gastroenterol Hepatol.

[4] Longato L (2013). Non-alcoholic fatty liver disease (NAFLD): a tale of fat and sugar?. Fibrogenesis Tissue Repair.

[5] Zarfeshani A, Ngo S, Sheppard AM (2015). MicroRNA expression relating to dietary-induced liver steatosis and NASH. J Clin Med.

[6] Arefhosseini SR, Ebrahimi-Mameghani M, Farsad Naeimi A, Khoshbaten M, Rashid J (2011). Lifestyle modification through dietary intervention: health promotion of patients with non-alcoholic fatty liver disease. Health Promot Perspect.

[7] Starley BQ, Calcagno CJ, Harrison SA (2010). Nonalcoholic fatty liver disease and hepatocellular carcinoma: a weighty connection. Hepatology.

[8] Marra F, Gastaldelli A, Svegliati Baroni G, Tell G, Tiribelli C (2008). Molecular basis and mechanisms of progression of non-alcoholic steatohepatitis. Trends Mol Med.

[9] Khavasi N, Somi MH, Khadem E, Faramarzi E, Ayati MH, Fazljou SMB (2017). Effect of daily caper fruit pickle consumption on disease regression in patients with non-alcoholic fatty liver disease: a double-blinded randomized clinical trial. Adv Pharm Bull.

[10] Gori M, Arciello M, Balsano C (2014). MicroRNAs in nonalcoholic fatty liver disease: novel biomarkers and prognostic tools during the transition from steatosis to hepatocarcinoma. Biomed Res Int.

[11] Bartel DP (2004). MicroRNAs: genomics, biogenesis, mechanism, and function. Cell.

[12] Di Leva G, Garofalo M, Croce CM (2014). MicroRNAs in cancer. Annu Rev Pathol.

[13] Szabo G, Csak T (2016). Role of MicroRNAs in NAFLD/NASH. Dig Dis Sci.

[14] Heidary MF, Mahmoodzadeh Hosseini H, Mehdizadeh Aghdam E, Nourani MR, Ranjbar R, Mirnejad R (2015). Overexpression of metastatic related microRNAs, miR-335 and miR-10b, by staphylococcal enterotoxin B in the metastatic breast cancer cell line. Adv Pharm Bull.

[15] Ceccarelli S, Panera N, Gnani D, Nobili V (2013). Dual role of microRNAs in NAFLD. Int J Mol Sci.

[16] Baffy G (2015). MicroRNAs in nonalcoholic fatty liver disease. J Clin Med.

[17] Oseini AM, Sanyal AJ (2017). Therapies in non-alcoholic steatohepatitis (NASH). Liver Int.

[18] Karimi-Sales E, Mohaddes G, Alipour MR (2018). Chalcones as putative hepatoprotective agents: preclinical evidence and molecular mechanisms. Pharmacol Res.

[19] Karimi-Sales E, Jeddi S, Ebrahimi-Kalan A, Alipour MR (2018). trans-Chalcone prevents insulin resistance and hepatic inflammation and also promotes hepatic cholesterol efflux in high-fat diet-fed rats: modulation of miR-34a-, miR-451-, and miR-33a-related pathways. Food Funct.

[20] Karimi-Sales E, Ebrahimi-Kalan A, Alipour MR (2019). Preventive effect of trans-chalcone on non-alcoholic steatohepatitis: Improvement of hepatic lipid metabolism. Biomed Pharmacother.

[21] Karimi-Sales E, Jeddi S, Ebrahimi-Kalan A, Alipour MR (2018). Trans-chalcone enhances insulin sensitivity through the miR-34a/SIRT1 pathway. Iran J Basic Med Sci.

[22] Zou Y, Li J, Lu C, Wang J, Ge J, Huang Y (2006). High-fat emulsion-induced rat model of nonalcoholic steatohepatitis. Life Sci.

[23] Al-Daihan S, Shafi Bhat R (2015). Impact of propionic acid on liver damage in rats. Int J Mol Cell Med.

[24] Bonora E, Targher G, Alberiche M, Bonadonna RC, Saggiani F, Zenere MB (2000). Homeostasis model assessment closely mirrors the glucose clamp technique in the assessment of insulin sensitivity: studies in subjects with various degrees of glucose tolerance and insulin sensitivity. Diabetes Care.

[25] Dixon JB, Bhathal PS, O’Brien PE (2001). Nonalcoholic fatty liver disease: predictors of nonalcoholic steatohepatitis and liver fibrosis in the severely obese. Gastroenterology.

[26] Najafian M, Ebrahim-Habibi A, Yaghmaei P, Parivar K, Larijani B (2010). Core structure of flavonoids precursor as an antihyperglycemic and antihyperlipidemic agent: an in vivo study in rats. Acta Biochim Pol.

[27] Cheung O, Puri P, Eicken C, Contos MJ, Mirshahi F, Maher JW (2008). Nonalcoholic steatohepatitis is associated with altered hepatic microRNA expression. Hepatology.

[28] Jopling C (2012). Liver-specific microRNA-122: Biogenesis and function. RNA Biol.

[29] Wen J, Friedman JR (2012). miR-122 regulates hepatic lipid metabolism and tumor suppression. J Clin Invest.

[30] Yang YM, Seo SY, Kim TH, Kim SG (2012). Decrease of microRNA-122 causes hepatic insulin resistance by inducing protein tyrosine phosphatase 1B, which is reversed by licorice flavonoid. Hepatology.

[31] Su D, Zhang R, Hou F, Chi J, Huang F, Yan S (2017). Lychee pulp phenolics ameliorate hepatic lipid accumulation by reducing miR-33 and miR-122 expression in mice fed a high-fat diet. Food Funct.

[32] Baselga-Escudero L, Arola-Arnal A, Pascual-Serrano A, Ribas-Latre A, Casanova E, Salvadó MJ (2013). Chronic administration of proanthocyanidins or docosahexaenoic acid reverses the increase of miR-33a and miR-122 in dyslipidemic obese rats. PLoS One.

[33] Blasco-Baque V, Coupé B, Fabre A, Handgraaf S, Gourdy P, Arnal JF (2017). Associations between hepatic miRNA expression, liver triacylglycerols and gut microbiota during metabolic adaptation to high-fat diet in mice. Diabetologia.

[34] Ahn J, Lee H, Jung CH, Ha T (2012). Lycopene inhibits hepatic steatosis via microRNA-21-induced downregulation of fatty acid-binding protein 7 in mice fed a high-fat diet. Mol Nutr Food Res.

[35] Loyer X, Paradis V, Hénique C, Vion AC, Colnot N, Guerin CL (2016). Liver microRNA-21 is overexpressed in non-alcoholic steatohepatitis and contributes to the disease in experimental models by inhibiting PPARα expression. Gut.

[36] Kong L, Zhu J, Han W, Jiang X, Xu M, Zhao Y (2011). Significance of serum microRNAs in pre-diabetes and newly diagnosed type 2 diabetes: a clinical study. Acta Diabetol.

[37] Choi SE, Fu T, Seok S, Kim DH, Yu E, Lee KW (2013). Elevated microRNA-34a in obesity reduces NAD+ levels and SIRT1 activity by directly targeting NAMPT. Aging Cell.

[38] Choi SE, Kemper JK (2013). Regulation of SIRT1 by microRNAs. Mol Cells.

[39] Hur W, Lee JH, Kim SW, Kim JH, Bae SH, Kim M (2015). Downregulation of microRNA-451 in non-alcoholic steatohepatitis inhibits fatty acid-induced proinflammatory cytokine production through the AMPK/AKT pathway. Int J Biochem Cell Biol.

[40] Zhuo S, Yang M, Zhao Y, Chen X, Zhang F, Li N (2016). MicroRNA-451 negatively regulates hepatic glucose production and glucose homeostasis by targeting glycerol kinase-mediated gluconeogenesis. Diabetes.

